# Relationship between the longitudinal trajectory of the triglyceride-glucose index and the development of CKD: an 8-year retrospective longitudinal cohort study

**DOI:** 10.3389/fendo.2024.1376166

**Published:** 2024-05-15

**Authors:** Qinchuan Hou, Huiwang Zhang, Rui Zhang, Binghong Li, Lei Li, Dongyu Li, Xian Wang, Yuping Liu, Zhengwei Wan, Junlin Zhang, Ping Shuai

**Affiliations:** ^1^ Health Management Center & Health Management Research Institute, Sichuan Provincial People’s Hospital, Chengdu, China; ^2^ School of Public Health, Southwest Medical University, Luzhou, China; ^3^ School of Medicine, University of Electronic Science and Technology of China, Chengdu, China

**Keywords:** triglyceride-glucose index, change trajectory, chronic kidney disease, health check-up population, cohort study

## Abstract

**Background:**

The triglyceride-glucose (TyG) index, a simple surrogate marker of insulin resistance, is significantly associated with chronic kidney disease (CKD). However, there is limited research on the longitudinal trajectory of TyG index over time and its relationship with CKD.

**Objective:**

To analyse the characteristics of the longitudinal trajectory of the TyG index over time and its association with the development of CKD in a health check-up population.

**Methods:**

Participants who underwent at least three annual health check-ups at the Health Management Center of Sichuan Provincial People’s Hospital from 2015 to 2022 were included in this retrospective cohort study. The TyG index was calculated as ln [fasting triglycerides (mg/dL) × fasting glucose (mg/dL)/2]. The latent class mixed model (LCMM) was used to identify the TyG index trajectory of the study population. A Cox proportional hazard model was used to estimate the CKD incidence risk in different quartile groups and the association of changes in the TyG index trajectory with the development of CKD.

**Results:**

A total of 4,921 participants were included in this study, and they were divided into four groups according to the quartiles of the baseline TyG index: Q1 (5.43-6.66), Q2 (6.67-7.04), Q3 (7.05-7.43), and Q4 (7.43-9.97). There was no difference in the risk of CKD occurrence among the TyG groups. Three different TyG index trajectories were identified in this study: a high-level group, middle-level stable group and low-level stable group, respectively. The incidence rate of CKD in the high-level TyG index trajectory group was 2.399 times greater than that in the low-level stable trajectory group (HR=2.399, 95% CI 1.167-4.935).

**Conclusion:**

Individuals with long-term exposure to high TyG index levels had a significantly greater risk of CKD. Routine monitoring of the TyG index and its longitudinal trend will aid in the risk stratification of CKD in the general population and will be helpful for CKD prevention and targeted management.

## Introduction

CKD is a noncommunicable disease that ranks eleventh in global mortality. Current data indicate that the global incidence of CKD ranges from approximately 8%-16% (1). Given its irreversible progression, the burden of CKD has increased rapidly, and CKD has the highest disability and mortality among chronic diseases (2–4). The onset of CKD is often asymptomatic due to the strong compensatory mechanism of the kidneys. Additionally, the absence of sensitive and specific biomarkers presents challenges for the early diagnosis and prevention of CKD (5). Thus, there is a crucial need to identify simple, efficient and applicable screening indicators for the early detection of CKD in clinical practice.

Recent studies have shown that patients with CKD frequently exhibit insulin resistance (IR), which is a critical risk factor for disease progression (6). The gold standard for measuring IR is the hyperinsulinemia/euglycemic clamp technique, which is relatively expensive and difficult to perform (7). Currently, the homeostasis model assessment of insulin resistance (HOMA-IR) is recognized as a simple and accurate indicator of IR, requiring the measurement of fasting insulin. However, the examination of fasting insulin is difficult in most primary hospitals (8–10). The TyG index, as a new alternative indicator of IR, is calculated simply using only the two indices of fasting blood glucose and triglycerides, which can be easily obtained and monitored in the clinic (11–15). Previous cross-sectional studies have indicated that the baseline TyG index is independently correlated with the incidence of CKD, and some studies have demonstrated its association with CKD progression (16–20). However, the long-term effects of the TyG index and its trend over time on CKD have seldom been investigated.

Currently, trajectory analysis methods are being increasingly applied to the study of different diseases, such as hypertension and cardiovascular diseases (21–23). Thus, in this study, we aimed to determine how the trajectory characteristics of the TyG index change over time and sought to identify the relationship of the TyG index with the onset of CKD in the health check-up population, hoping to provide evidence on the association of TyG index trends with CKD incidence.

## Materials and methods

### Study participants

**Figure 1:**
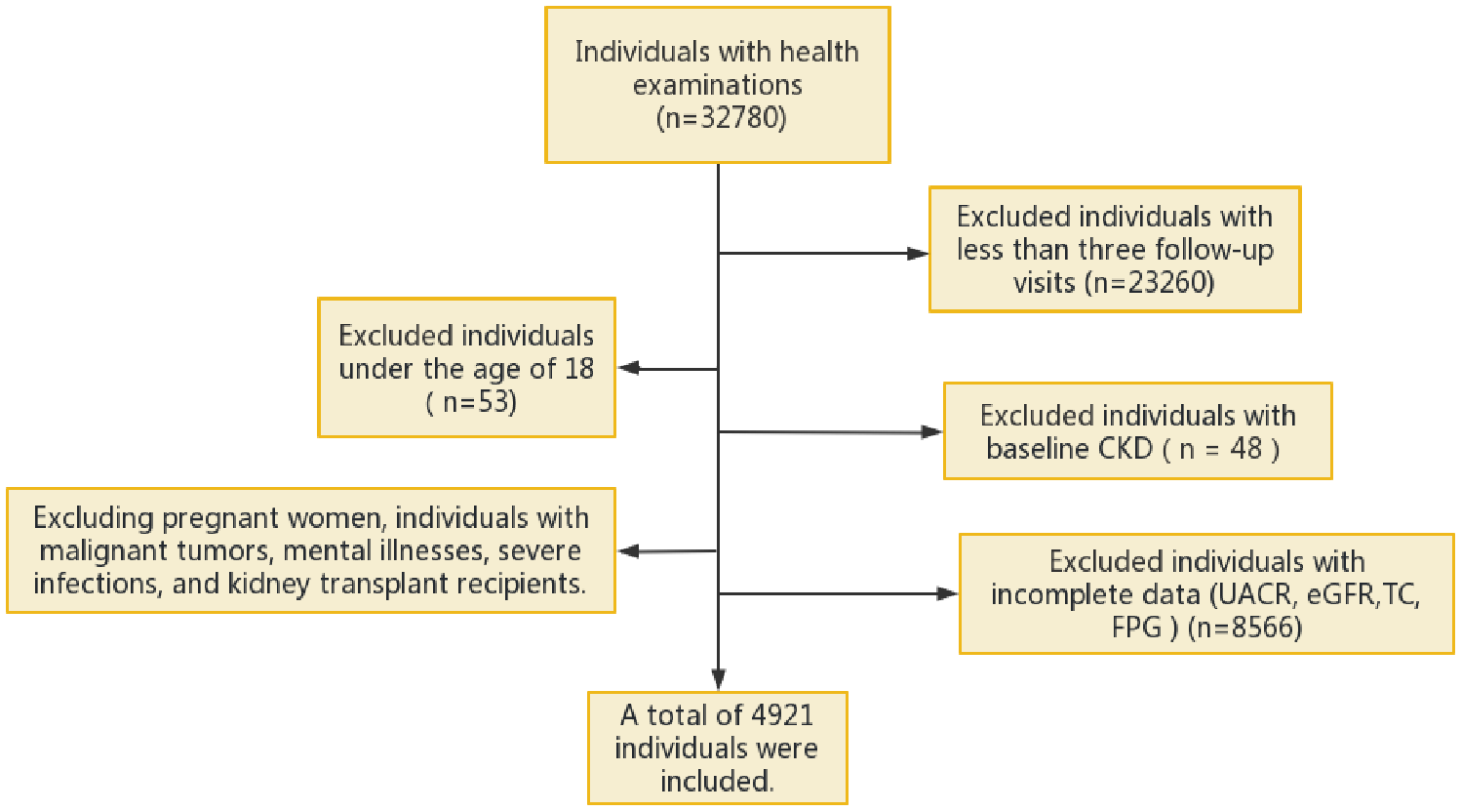
Flowchart of the inclusion process of the study. eGFR, estimated glomerular filtration rate; UACR, urine albumin-creatinine ratio; FPG, fasting plasma glucose; TC, total cholesterol; CKD, chronic kidney disease.

This was a retrospective cohort study in which individuals who had undergone annual health examinations at the Health Management Center of Sichuan Provincial People’s Hospital from January 2015 to November 2022 were selected. The inclusion and exclusion criteria are detailed in **Figure 1**. The study included adult participants who had undergone at least three health examination surveys and who had contributed complete data on estimated glomerular filtration rate (eGFR), the urine albumin−creatinine ratio (UACR), fasting plasma glucose (FPG), and triglyceride (TG) levels. Individuals with liver cirrhosis, severe infections, malignant tumours, or mental disorders, and those who underwent haemodialysis or peritoneal dialysis, who underwent kidney transplantation, who displayed CKD, or who had an abnormal UACR or eGFR at baseline, were excluded.

### Data collection

Basic information (name, sex, age), lifestyle and behaviour (exercise habits, smoking history, alcohol consumption), and personal medical history (such as hypertension, hyperlipidaemia, diabetes, liver cirrhosis, and tumour history) of the study participants were collected using a standardized questionnaire. A professional nurse measured the waist circumference of each participant after they sat quietly, and their sitting blood pressure was measured three times using an Omron HBP-9020 fully automatic electronic sphygmomanometer, with the average value taken as the final value. The height and weight of the participants were measured using an SK-14B0121 electronic scale (Shenzhen Shuangjia Electronics Co., Ltd.), and body mass index (BMI) was calculated as BMI = body weight (kg)/height (m)^2^.

Venous blood samples were collected from the participants in a fasting state in the morning, and a fully automated biochemical analyser was used to measure TG, total cholesterol (TC), high-density lipoprotein cholesterol (HDL-C), low-density lipoprotein cholesterol (LDL-C), serum creatinine (SCr), uric acid (UA), FPG, and glycosylated haemoglobin (HbA1c) levels. The TyG index was calculated as TyG index = ln[TG (mg/dL) × FPG (mg/dL)/2] (24).

Urine specimens were collected from the participants, and analysed using a fully automated biochemical analyser to measure urine albumin and urine creatinine. Subsequently, UACR and eGFR were calculated, where UACR = urine albumin (mg/L)/urine creatinine (g/L), and eGFR was calculated using the 2019 Chronic Kidney Disease Epidemiology Collaboration equation (CKD-EPI) (25).

### Definitions

The primary outcome measure was the incidence of CKD, which was defined as eGFR<60 mL·min^-1^·(1.73 m^2^)^-1^ and/or UACR≥30mg/g for at least 3 months according to the KDIGO guidelines (26). Patients with HbA1c ≥6.5% and patients with previously diagnosed diabetes mellitus were diagnosed with DM (27). Hypertension was diagnosed as a systolic blood pressure (SBP) ≥140 and/or diastolic blood pressure (DBP) ≥90 mm Hg or a previous history of hypertension or current use of antihypertensive medication (28). A TC concentration ≥5.18 mmol/L, a TG concentration ≥1.70 mmol/L, an LDL-C concentration ≥3.37 mmol/L, an HDL-C concentration <1.04 mmol/L, and/or the current use of lipid-regulating drugs all indicate the presence of dyslipidaemia (29). Frequent smoking was defined as smoking, and the rest as non-smoking. Frequent drinking was defined as drinking, and the rest as non-drinking.

### Statistical methods

At baseline, the characteristics of the study subjects were described according to quartiles of TyG index levels. In the longitudinal analysis, the subjects’ characteristics were described based on the TyG index trajectories. Normally distributed metric data are expressed as the mean ± standard deviation (χ±SD), and between-group comparisons were performed using t tests or analysis of variance (ANOVA), as appropriate. Non-normally distributed metric data are represented as medians with interquartile ranges (IQRs). Count data are expressed as counts with percentages, and between-group comparisons were performed using the chi-square test.

A LCMM was used to identify different developmental patterns of changes in the TyG index. The TyG index trajectory was set as a polynomial function of follow-up time. Linear, quadratic, and cubic forms of the polynomial function were explored. To avoid a low proportion of individuals in each latent class, each form was fitted with 1-5 groups. The optimal model was selected based on the following criteria: (1) lower Bayesian information criterion (BIC) values were preferred. (2) The average posterior probability of each class was greater than 0.7. (3) The proportion of individuals with high posterior probabilities (>0.7) in each class exceeded 65% (30, 31).

Cox proportional hazards regression models were used to analyse the relationships between quartiles of the TyG index and CKD, the TyG index trajectory and CKD, respectively. Restricted cubic spline regression (RCS) was used to analyse the dose−response relationship between the TyG index and CKD occurrence risk and to determine the appropriate TyG cut-off point. Two multivariate models were used to adjust for potential confounding factors for CKD occurrence. Model 1 was adjusted for baseline age and sex; Model 2 was further adjusted for smoking status, alcohol consumption status, hypertension status, BMI, and UA.

The data were analysed using SPSS software (version 26.0) and R software (version 4.2.2). P<0.05 was considered to indicate statistical significance.

## Results

### Baseline characteristics of participants by quartiles of the TyG index

**Figure 2:**
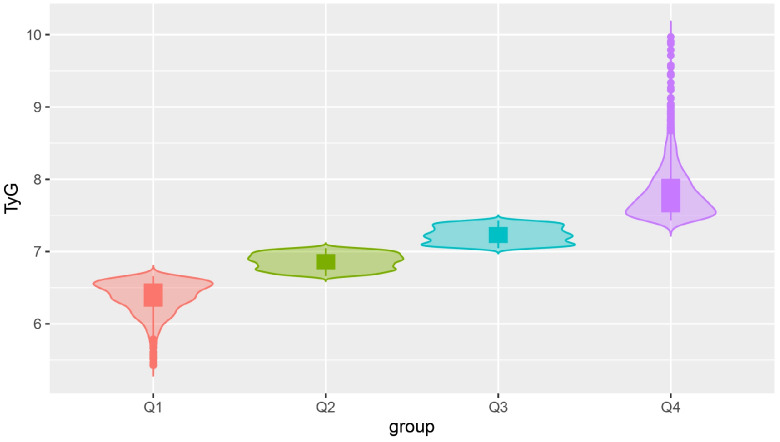
Violin plot of the TyG index quartiles. TyG, the triglyceride-glucose index.

The present study included 4,921 participants in the cohort, 3,139 (63.79%) men and 1,782 (36.21%) women. The mean age was 54.58±12.39 years and the mean TyG index was 7.08±0.59. According to the quartiles of the baseline TyG index, the subjects were divided into four groups: Q1 (5.43-6.66), Q2 (6.67-7.04), Q3 (7.05-7.43), and Q4 (7.43-9.97), as illustrated in **Figure 2**. The proportion of the males and the levels of SBP, DBP, BMI, TC, TG, FBG, HbA1c, and UA in the Q4 group were significantly higher compared with those in the other three groups. In addition, the frequencies of smoking and drinking, as well as the prevalence of hypertension, diabetes, and hyperlipidaemia were also higher in the Q4 group than those in the other groups (all *P* values were <0.05), as shown in **Table 1**.

**Table 1:** Characteristics of participants by the quartiles of TyG index in baseline (n=4921).

Variables	All	Quartiles of TyG index				P
		Q1(5.43~6.66, n=1230)	Q2 (6.67~7.04, n=1231)	Q3 (7.05~7.43, n=1230)	Q4(7.43~9.97, n=1230)	
Age, years	54.585±12.39	52.25±13.00	55.42±12.74	55.89±12.00	54.76±11.46	<0.001
Male, n (%)	3139 (63.8)	558 (45.4)	770 (62.6)	827 (67.2)	984 (80.0)	<0.001
Smoker, n (%)	839 (17.0)	115 (9.8)	163 (13.9)	221 (18.9)	340 (28.9)	<0.001
Drinker, n (%)	686 (13.9)	93 (8.0)	144 (12.3)	171 (14.6)	278 (23.6)	<0.001
Hypertension, n (%)	903 (18.3)	140 (11.4)	211 (17.1)	265 (21.5)	287 (23.3)	<0.001
Diabetes, n (%)	479 (9.7)	54 (4.4)	81 (6.6)	118 (9.6)	226 (18.4)	<0.001
Hyperlipidemia, n (%)	356 (7.2)	44 (3.6)	72 (5.9)	98 (8.0)	142 (11.5)	<0.001
SBP, mmHg	121.64±16.47	116.73±16.06	121.17±16.45	122.97±15.97	125.69±16.11	<0.001
DBP, mmHg	72.70±10.63	69.12±9.89	72.07±10.51	73.62±10.37	76.00±10.59	<0.001
BMI,kg/m2	23.98±2.99	22.38±2.70	23.63±2.77	24.53±2.86	25.37±2.78	<0.001
TC, mmol/L	4.86±0.91	4.54±0.83	4.80±0.89	4.96±0.88	5.14±0.92	<0.001
LDL-C, mmol/L	2.85±0.98	2.57±0.69	2.90±0.79	3.04±0.79	2.89±0.82	<0.001
TG, mmol/L	1.67±1.19	0.78±0.17	1.20±0.19	1.68±0.29	3.02±1.64	<0.001
HDL-C, mmol/L	1.32±0.33	1.54±0.33	1.37±0.30	1.26±0.26	1.11±0.24	<0.001
FBG, mmol/L	5.20±1.11	4.78±0.54	5.00±0.65	5.21±0.80	5.81±1.72	<0.001
HbA1c,%	5.59±0.69	5.38±0.44	5.51±0.49	5.61±0.58	5.88±1.00	<0.001
UA,umol/L	350.34±84.51	310.20±75.80	340.55±79.83	360.33±76.40	390.27±85.03	<0.001
TyG index	7.08±0.59	6.37±0.23	6.86±0.11	7.23±0.11	7.84±0.39	<0.001

SBP, systolic blood pressure; DBP, diastolic blood pressure; BMI, body mass index; TC, total cholesterol; LDL-C, low-density lipoprotein cholesterol; TG, triglycerides; HDL-C, high-density lipoprotein cholesterol; FPG, fasting plasma glucose; HbA1c, glycosylated hemoglobin; UA, uric acid.

### Association of the TyG index with CKD occurrence

At the end of the 8-year follow-up, 139 participants developed CKD. The incidences of CKD in the Q1-Q4 groups were 1.79% (22), 2.68% (33), 2.68% (33), and 4.15% (51). Cox regression model (**Table 2**) showed that there was a statistically significant difference in the risk of CKD between the Q4 and the Q1 group, both under the unadjusted model without confounding variables and the adjusted model for the age and sex. After adjusted for the following more factors, which included the BMI, UA, smoking and alcohol consumption status, diabetes history, hyperlipidaemia history and hypertension history, no significant difference was detected in the risk of CKD between the Q1 and the other three quantile groups (P>0.05).

**Table 2:** Association of the TyG index with CKD incidence in the 8-year follow-ups (n=4921).

TyG index	Unadjusted		Model 1		Model 2	
	HR (95% CI)	P value	HR (95% CI)	P value	HR (95% CI)	P value
Q1 (5.4-6.7)	Reference		Reference		Reference	
Q2 (6.7-7.0)	1.388 (0.809, 2.381)	0.234	1.242 (0.723, 2.133)	0.433	1.145 (0.647, 2.026)	0.642
Q3 (7.0~ 7.4)	1.340 (0.781, 2.299)	0.289	1.243 (0.718, 2.210)	0.447	0.985 (0.552, 1.755)	0.958
Q4 (7.4–10.0)	2.243 (1.360, 3.698)	**0.002**	2.210 (1.337, 3.653)	**0.002**	1.257 (0.716, 2.205)	0.425

Model 1, adjusted for gender and age.

Model 2, further adjusted for smoking, alcohol drinking, diabetes, hypertension, hyperlipidemia, BMI, UA.

Boldface indicates values with a p-value<0.05.

### Dose−response relationship between the TyG index and the risk of CKD

**Figure 3:**
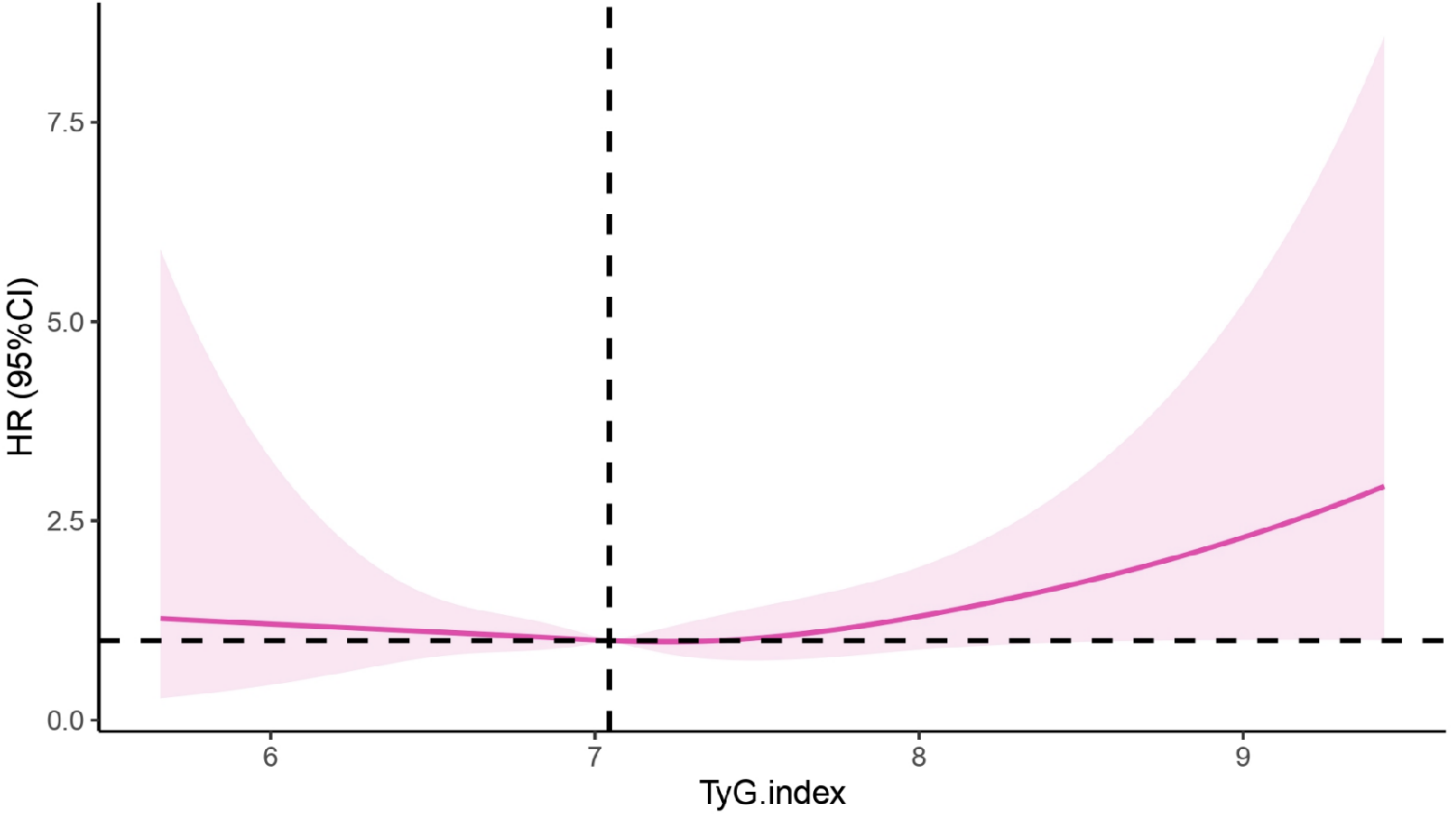
Correlation between the TyG index and CKD based on the RCS. CKD, chronic kidney disease; TyG, the triglyceride-glucose index; HR, hazard ratio.

In this study, the RCS was further used to visualize the relationship between the TyG index and the risk of CKD. After adjusting for age, sex, BMI, UA, smoking and alcohol consumption status, disease histories, a linear correlation was revealed (P_nonlinearity_=0.325, knot=4). The incidence of CKD gradually decreased at first, but after the TyG index reached 7.05 or higher, the risk of CKD apparently increased with the increasing TyG index, as shown in **Figure 3**.

### Baseline characteristics of participants by TyG index trajectory

**Figure 4:**
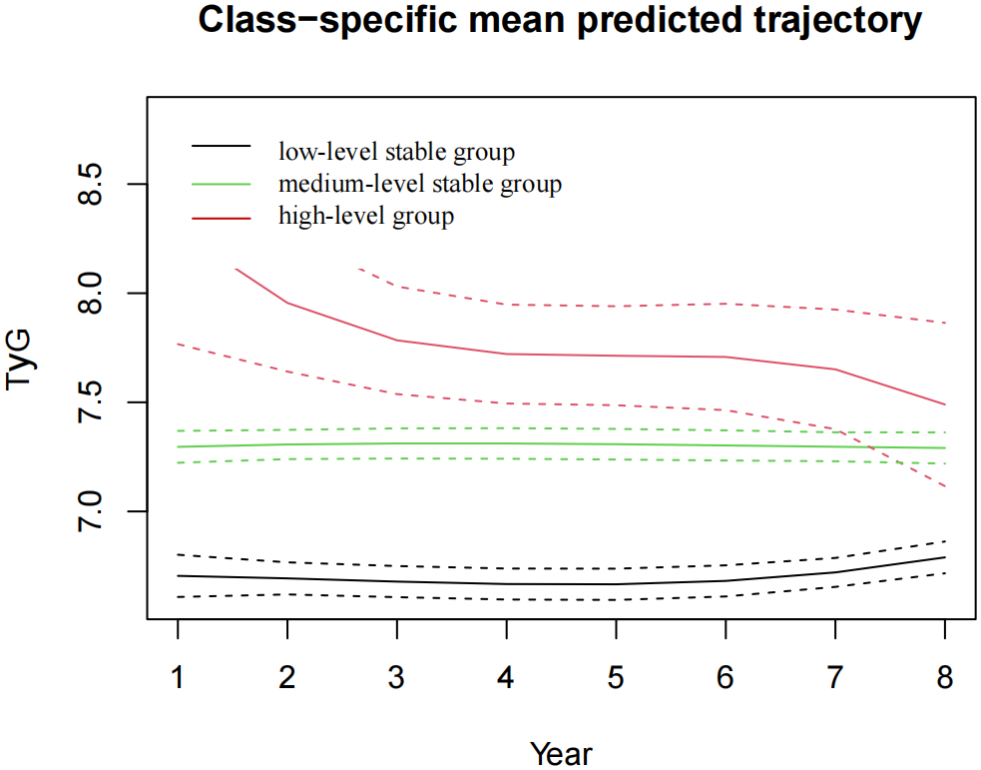
The TyG index trajectory groups in the 8-year follow-up. TyG, the triglyceride-glucose index.

The pattern of change in the TyG index of this population was analysed from 2015 to 2022. We identified three different trajectories of the TyG index, which were the low-level stable group (n=2,101, 42.69%), the medium-level stable group (n=2,692, 54.7%) and the high-level stable group (n=128, 2.6%), as shown in **Figure 4**. At baseline, the TyG index values for each dynamic trajectory group were 6.59±0.33, 7.39±0.39, and 8.39±0.79, respectively (**Table 3**). Compared with the low-level stable group, the medium-level stable group and high-level stable group had greater percentages of males; higher levels of SBP, DBP, BMI, TC, TG, FBG, HbA1c and UA; higher frequencies of smoking and alcohol consumption; as well as greater prevalence of hypertension, diabetes, and dyslipidaemia (P<0.05 for all).

**Table 3:** Baseline characteristics of participants by the TyG index trajectory groups (n=4921).

Variables	All	Trajectory groups of the TyG index			P
		Low-level stable group (n=2101)	Middle-level stable group (n=2692)	High-level group (n=128)	
Male, n (%)	3139 (63.8)	1069 (50.8)	1964 (73.0)	106 (82.8)	<0.001
Age, years	54.585±12.39	53.49±12.99	55.48±11.91	53.47±10.71	<0.001
Smoker, n (%)	839 (17.0)	224 (11.2)	566 (22.1)	49 (40.2)	<0.001
Drinker, n (%)	686 (13.9)	184 (9.2)	475 (18.5)	27 (22.1)	<0.001
Hypertension, n (%)	903 (18.3)	279 (13.3)	592 (22.0)	32 (25.0)	<0.001
Diabetes, n (%)	479 (9.7)	101 (4.81)	325 (12.07)	53 (41.41)	<0.001
Hyperlipidemia, n (%)	356 (7.2)	88 (4.2)	252 (9.4)	16 (12.5)	<0.001
SBP, mmHg	121.64±16.47	118.31±16.19	124.14±16.24	123.66±16.46	<0.001
DBP, mmHg	72.70±10.63	70.26±10.17	74.45±10.63	75.96±10.12	<0.001
BMI, kg/m2	23.98±2.99	22.79±2.78	24.84±2.82	25.39±2.97	<0.001
TC, mmol/L	4.86±0.91	4.65±0.85	5.00±0.91	5.29±0.98	<0.001
LDL-C, mmol/L	2.85±0.98	2.70±0.74	2.99±0.81	2.48±0.83	<0.001
TG, mmol/L	1.67±1.19	0.98±0.31	2.04±0.86	5.29±3.72	<0.001
HDL-C, mmol/L	1.32±0.33	1.48±0.33	1.21±0.26	1.00±0.26	<0.001
FBG, mmol/L	5.20±1.11	4.87±0.58	5.37±1.11	6.98±3.17	<0.001
HbA1c,%	5.59±0.69	5.43±0.45	5.68±0.73	6.45±1.62	<0.001
UA, umol/L	350.34±84.51	319.99±78.14	372.36±81.37	385.35±93.27	<0.001
TyG index	7.08±0.59	6.59±0.33	7.39±0.39	8.39±0.79	<0.001

SBP, systolic blood pressure; DBP, diastolic blood pressure; BMI, body mass index; TC, total cholesterol; LDL-C, low-density lipoprotein cholesterol; TG, triglycerides; HDL-C, high-density lipoprotein cholesterol; FPG, fasting plasma glucose; HbA1c, glycosylated hemoglobin; UA, uric acid.

### Association of the TyG index trajectory with CKD occurrence

After 8 years of follow-up, the incidences of CKD in the low-level stable group, medium-level stable group, and high-level stable group were1.8% (38), 3.3% (90), and 8.6% (11), respectively. Univariate Cox regression analysis revealed that the risk of incident CKD in the high-level group was 4.497 times greater than that in the low-level stable group (hazard ratio (HR) =4.497, 95% CI 2.298-8.798). After adjusting for covariates such as age, sex, BMI, UA, smoking and alcohol consumption status, as well as disease histories, the risk of CKD in the high-level group was 2.399 times greater than that in the low-level stable group (HR=2.399, 95% CI 1.167-4.935), with P<0.05, as shown in **Table 4**.

**Table 4:** Association of the TyG index trajectories with CKD incidence in the 8-year follow-ups (n=4921).

TyG index	Unadjusted		Model 1		Model 2	
	HR (95% CI)	P value	HR (95% CI)	P value	HR(95% CI)	P value
Low-level stable group	Reference		Reference		Reference	
Middle-level stable group	1.714 (1.172,2.505)	**0.005**	1.700 (1.162,2.487)	**0.006**	1.196 (0.785,1.821)	0.404
High-level group	4.497 (2.298,8.798)	**<0.001**	5.145 (2.623,10.092)	**<0.001**	2.399 (1.167,4.935)	**0.017**

Model 1, adjusted for gender and age.

Model 2, further adjusted for smoking, alcohol drinking, diabetes, hypertension, hyperlipidemia, BMI, UA.

Boldface indicates values with a p-value<0.05.

### Subgroup analysis of the risk of CKD occurrence

**Figure 5:**
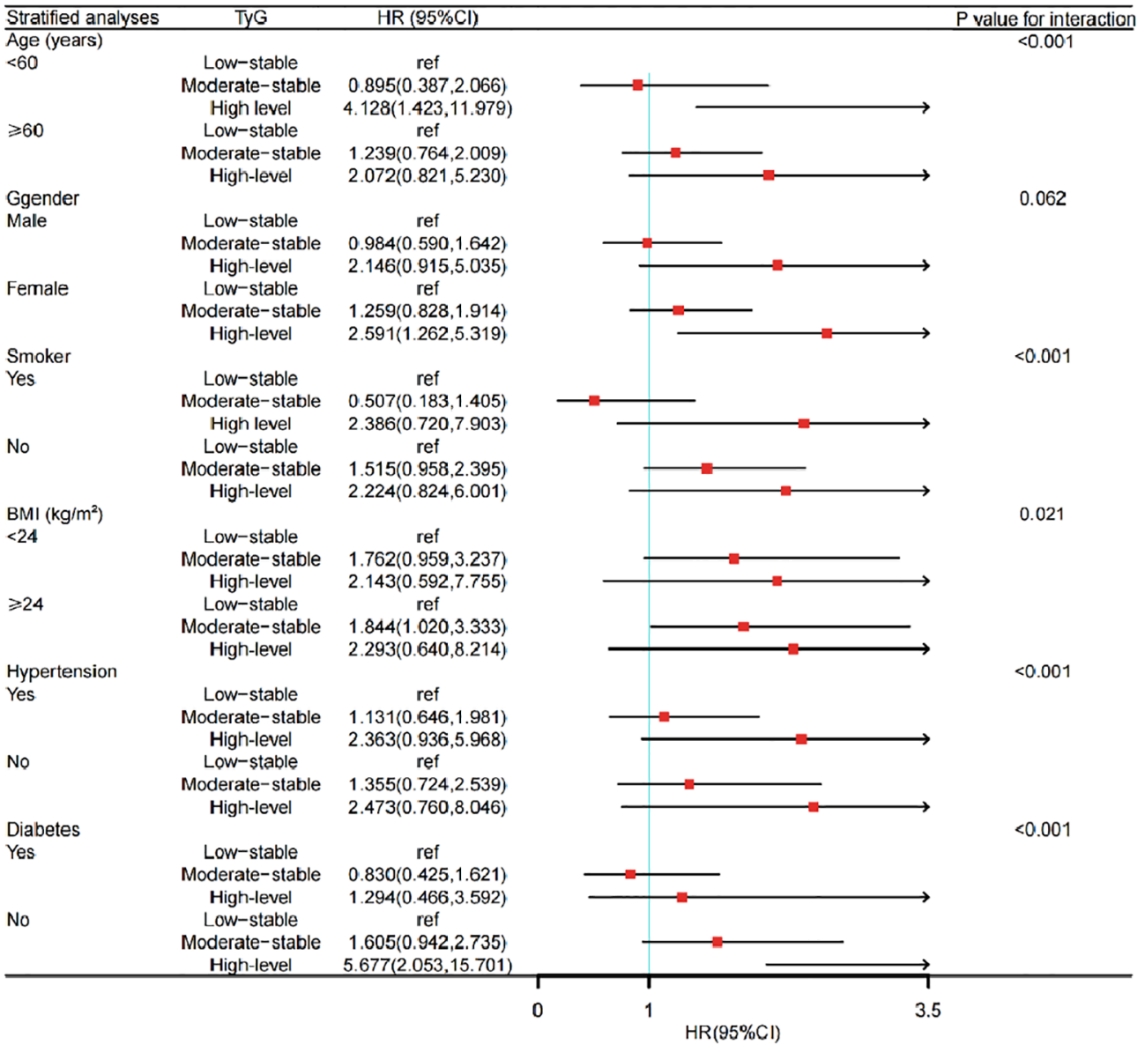
Subgroup analysis of the impact of TyG trajectory groups on risk of CKD occurrence. CKD, chronic kidney disease; TyG, the triglyceride-glucose index; HR, hazard ratio.

Subgroup analysis showed that there were obvious correlations between the TyG index trajectory and the risk of CKD in different sex, age, BMI level and diabetes history groups. Among the people with BMI over than 24 kg/m^2^, the incidence of CKD in the moderate-level stable group was increased, compared with that in the low-level stable group. Similarly, among the female population, those older than 60 years, and non-diabetes, the risk of CKD was significantly higher in the high-level stable group, as shown in **Figure 5**.

## Discussion

In this study, we observed a significant correlation between the TyG index and CKD incidence. At baseline, when the patients were divided into four groups based on quartiles, no difference of the risk of CKD was seen among the remaining three groups compared to the Q1 group. 8 years later, three distinct subgroups were identified based on the initial level and trajectory characteristics of the TyG index, which were the low-level stable group, the medium-level stable group, and the high-level group. Compared with the low-level stable group, the high-level group had a significantly increased risk of developing CKD. This suggests that the TyG index is closely correlated with the development of CKD and that high levels of IR and long-term exposure to high levels of IR may play an important role in the pathogenesis of CKD.

Increasing evidence indicates that the TyG index can be a reliable predictor of cardiovascular disease (32–34). Recently, attention has been directed to the relationship between the TyG index and CKD. This study revealed that at baseline, when patients were divided into four groups based on quartiles, there was no difference in the risk of developing CKD among the remaining three groups compared to the Q1 group. In this study, the baseline TyG index revealed no significant difference for the risk of CKD occurrence when compared with the quantile groups. The RCS model suggested that 7.05 was the cut-off value of the TyG index in this population. That is when the TyG index was less than 7.05, the risk of developing CKD decreased gradually. But when the TyG index was equal to 7.05 or greater, the risk was increased accordingly. Inconsistent results were found from a study in Xinjiang region of China, which revealed that the risk of CKD in the Q4 group was 1.63 times greater than that in Q1 group (95% CI: 1.14-2.33, P = 0.007). In this study, the relationship between the TyG index and CKD was nonlinear (P_nonlinear_ = 0.021) and the hazard ratio of CKD decreased first, then increased rapidly when the value of TyG index reached 8.94 approximately (35). The reason for these differences may be the objectives in our study were from a health check-up population, which involved a larger proportion of healthier people in the data sample. But the subjects in Xinjiang study were hypertension patients with abnormal blood glucose, who had an overall higher glucose level.

Several previous researches verified the association of the TyG index and CKD by cross-sectional study design. Our longitudinal study further confirmed this correlation. Moreover, we extended these observations by identifying different trajectories of the TyG index. We found that the TyG index may fluctuate over time, and a single point measurement cannot reflect the heterogeneity of trends adequately. The trajectory pattern of the TyG index provides additional information on the cumulative risk burden of CKD. Our results indicated that 8 years later, three distinct subgroups were identified based on the initial level and the trajectory characteristics of the TyG index, which were the low-level stable group, the medium-level stable group, and the high-level group. No statistically significant difference in the risk of developing CKD between the medium-level stable group and the low-level stable group. But there was a significant increase of the risk in the high-level group when compared with that in the low-level stable group. Although the TyG index in the high-level group showed a downwards trend during the follow-up period, it remained consistently much higher than that both in the low-level and medium-level stable groups, which resulting in a greater risk of CKD occurrence. We concluded that the TyG index was closely correlated with the development of CKD and the long-term exposure to the high level of the index significantly increased the risk of CKD. Regular monitoring of the TyG index, early intervention and treatment for IR are meaningful for CKD control. For only 139 (2.8%) new CKD patients were followed in this study, most of the subgroup analysis provided no significant results due to the limited sample size when being divided into different sub-group and due to the interaction effects.

The potential mechanism underlying the correlation between the TyG index and CKD may come from the IR. IR is an independent risk factor for decreased kidney function in the elderly individuals and occurs in the early stage of CKD (36). Insulin plays an important role in regulating water, balance of electrolyte and acid−base, and regulating arterial blood pressure in the kidney. IR is associated with the occurrence and progression of various kidney diseases. On the one hand, IR enhances the production of insulin-like growth factor-1 (IGF-1) by stimulating IGF-1 receptors, which stimulates the proliferation of vascular smooth muscle cells, induces the growth of mesangial cells, inhibits cell apoptosis, and finally leads to renal fibrosis. Furthermore, IR increases the sodium retention of tubules, promotes the salt sensitivity of blood pressure and intraglomerular pressure, which causes microalbuminuria and ultimately, leads to kidney damage (37). CKD can also increase the occurrence of insulin resistance, which finally forms a vicious circle by interacting with each other (38).

Our limitation indicated that this was a single-centre retrospective study with limited representativeness, for the participants were all from the health check-up population, and few data was provided for the intakes of protein and sodium which were critical on the pathogenesis of CKD. Therefore, in the future, multi-centre prospective studies with larger population are needed for further exploration on the relationship of the TyG index and CKD.

## Conclusion

Individuals with long-term exposure to high TyG index levels may have a greater risk of developing CKD. Regular monitoring of the TyG index may help identify individuals at high risk for CKD and be helpful for the early screening and CKD prevention.

## Data availability statement

The raw data supporting the conclusions of this article will be made available by the authors, without undue reservation.

## Ethics statement

The studies involving humans were approved by Sichuan Provincial Academy of Medical Sciences-Sichuan Provincial People’s Hospital. The studies were conducted in accordance with the local legislation and institutional requirements. The ethics committee/institutional review board waived the requirement of written informed consent for participation from the participants or the participants’ legal guardians/next of kin because This study is a retrospective cohort study and is exempt from obtaining informed consent from patients.

## Author contributions

QH: Writing – original draft. HZ: Writing – original draft. RZ: Writing – original draft. BL: Writing – original draft. LL: Writing – original draft. DL: Writing – review & editing. XW: Writing – review & editing. YL: Writing – review & editing. ZW: Writing – review & editing. JZ: Writing – review & editing. PS: Writing – original draft, Writing – review & editing.
