# Corrigendum: Relationship between the longitudinal trajectory of the triglyceride-glucose index and the development of CKD: an 8-year retrospective longitudinal cohort study

**DOI:** 10.3389/fendo.2024.1433515

**Published:** 2024-05-27

**Authors:** Qinchuan Hou, Huiwang Zhang, Rui Zhang, Binghong Li, Lei Li, Dongyu Li, Xian Wang, Yuping Liu, Zhengwei Wan, Junlin Zhang, Ping Shuai

**Affiliations:** ^1^ Health Management Center & Health Management Research Institute, Sichuan Provincial People’s Hospital, Chengdu, China; ^2^ School of Public Health, Southwest Medical University, Luzhou, China; ^3^ School of Medicine, University of Electronic Science and Technology of China, Chengdu, China

**Keywords:** triglyceride-glucose index, change trajectory, chronic kidney disease, health check-up population, cohort study

In the published article, there was an error in **Figure 4** as published. The red color indicates the high-level group and the green color indicates the medium-level stable group, which is now labeled the other way round. The corrected **Figure 4** and its caption appear below.

**Figure 4:**
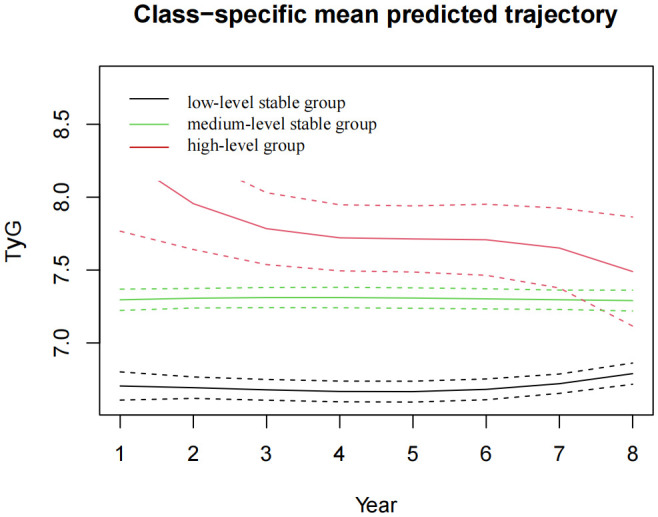
The TyG index trajectory groups in the 8-year follow-up. TyG, the triglyceride-glucose index.

The authors apologize for this error and state that this does not change the scientific conclusions of the article in any way. The original article has been updated.

